# Perceptions of journal editors on the use of eponyms in anatomical publishing: the need for compromise

**DOI:** 10.1007/s12565-024-00789-z

**Published:** 2024-07-17

**Authors:** Nicholas Bacci, Erin Hutchinson, Beverley Kramer, Brendon Kurt Billings

**Affiliations:** School of Anatomical Sciences, Faculty of Health Sciences, University of the Witwatersrand, 2nd Floor, 7 York Road, Parktown, Johannesburg, 2193 South Africa

**Keywords:** Anatomy, Eponyms, Ethics, Publishing, Terminology

## Abstract

While arguments have been made both for and against the value of anatomical eponyms, limited research exists regarding their use by anatomists. As the editors of the principal international anatomy journals have purview and control of terminology in anatomical journals, their perceptions regarding the relevance, ethics and inclusivity of eponym use in anatomy and in publishing in anatomical journals were investigated. A validated, confidential and anonymized self-administered questionnaire which included open-ended questions was distributed to the 22 Editor-in-Chiefs/Senior Editors of anatomical journals. Of the 16 (73%) editors who responded to the survey, only 56% were aware that eponyms had been censured since the time of the 1895 *Nomina Anatomica.* The majority of responding editors found the use of Latin- and Greek-derived terms more valuable when communicating with students and peers, but also thought eponyms were acceptable in manuscripts submitted to their journals. Thirteen (81%) of the responding editors agreed that eponyms play a vital role in the history of anatomy, and some thought they were important for discussing bioethics concepts. However, 62.5% felt that there were valid reasons for their discontinuation. Half of the respondents did not consider the continued use of eponyms an ethical concern. Responding editors of anatomical journals prefer the use of Latin- and Greek- derived terms when interacting with other anatomists and students. However, the continued use of eponyms was seen as an important opportunity for discussion on the history and ethics of anatomy.

**Supplementary Information:**

The online version contains supplementary material available at 10.1007/s12565-024-00789-z.

## Introduction

The first texts describing human anatomy originated from Hippocrates and Aristotle between 460 and 322 B.C.E and were later added to by Galen between 130 and 201 C.E. and relied almost exclusively on the classical languages of Latin and Greek (Turmezei 2012; Burdan et al. 2016). While anatomical terminology is thus somewhat ancient, the introduction of eponymous terms into the anatomical nomenclature occurred relatively recently. Eponyms relating to the names of specific physicians and anatomists or even fictitious characters, became prominent in the sixteenth and seventeenth centuries (Sakai 2007), as a way of honoring the individuals who proposed new discoveries in anatomy or were associated with the first detailed account of a structure (Gest 2014). Some particularly well-known eponymous terms originate from anatomists such as Bartolomeo Eustachi, Gabriele Fallopio, Thomas Willis, Boë Sylvius and many more. Eponyms are thus said to function both as a naming convention and as a tool for historical recollection (Andrew et al. 2023). However, by using eponymous terms, the association with the function or description of the structure is lost (Fargen and Hoh 2014).

Concern regarding the application of eponyms in anatomy stems from the assignment of a non-descriptive or non-functional term to a structure, making the understanding for students more difficult and encouraging rote learning. Occasionally, the eponymous term is applied to more than one structure leading to ambiguity and confusion. In addition, eponymous terms have been erroneously applied, may carry the name of two separate individuals or may be misleading (Duque-Parra et al. 2006).

Since 1895, with the first publication of the *Nomina Anatomica* (His 1895), followed by the International Federation of Associations of Anatomists (IFAA) *Nomina Anatomica* (International Anatomical Nomenclature Committee, 1977) and subsequently in 1998 and again in 2019 with the updated *Terminologia Anatomica* (TA) (FIPAT 2019), a strong drive has existed among anatomists in discussion platforms, social media and/or certain publications (e.g. Whitworth and Matterson 2007; Jana et al. 2009; Fargen and Hoh 2014; Olry 2014a; ANATOMYINCLAY 2023), to establish a uniform unambiguous communication medium for anatomy. As part of this effort, eponymous terminology was discontinued by the IFAA terminology committee (Federative Committee on Anatomical Terminology 1998), although a column on eponymous terms was retained in the TA to provide for the identification of the structures which have been eponymously named (FIPAT 2019). Despite the continuation of the use of eponymous terms by clinicians and anatomists alike, resistance and debate exists in both the anatomy learning and clinical spaces with regard to the use of these terms in anatomy and in medical terminology (Organ 1991; Duque-Parra et al. 2006; Strous and Edelman 2007; Whitworth 2007; Whitworth and Matterson 2007; Jana et al. 2009; Fargen and Hoh 2014; Gest 2014; Olry 2014a, b; Duque-Parra et al. 2018; McNulty et al. 2021; de Carvalho et al. 2022). Overall, the devising and usage of eponyms in medical terminology (inclusive of anatomical eponyms) steadily increased in the mid to late 1900s, reaching a peak in 1991 and has since slowly declined (Thomas 2016). However, certain clinical fields, such as neurology, continue to experience consistent use of eponymous terms and have seen a rise in new eponyms in the last 30 years (Zheng and Gold 2020).

Over 7200 eponymous anatomical and medical terms are said to exist in textbooks and online resources (Stuart-Smith et al. 2022). In more recent times, most healthcare workers and students no longer study the classical languages of Latin and Greek outside of their exposure to anatomy and thus find the anatomical terminology difficult to learn and understand (Smith et al. 2007). Having to contend with non-descriptive eponymous terms in addition to the classical terminology with its mostly unfamiliar linguistic origin, increases the cognitive load on health sciences students (McNulty et al. 2021).

While teaching of etymology is recommended to aid in the understanding of anatomical terminology (Turmezei 2012), many anatomists and clinicians choose instead to use eponymous terminology as they abbreviate some of the more complex anatomical terms (Kachlik et al. 2008) and provide clinicians with a quicker and easy way of recording details (Organ and Mussell 2021). As anatomical terminology includes the eponyms of infamous historical individuals, their continued use also provides anatomists with a seminal opportunity to discuss both the history of anatomy (Hildebrandt 2022) as well as ethics (Organ and Mussell 2021) with students. Discussions of eponyms could also highlight disparities in the anatomical terminology with respect to the ongoing discussion of equity and inclusivity in the anatomical curriculum (Meyer and Cui 2019; Longhurst et al. 2024).

There is considerable opinion in the literature on the use of eponyms (Organ 1991; Duque-Parra et al. 2006; Strous and Edelman 2007; Whitworth 2007; Whitworth and Matterson 2007; Jana et al. 2009; Fargen and Hoh 2014; Gest 2014; Olry 2014a, b; Duque-Parra et al. 2018; McNulty et al. 2021; de Carvalho et al. 2022). However, limited evidence-based research has been conducted on eponym use (e.g. Winkelmann 2012; Thomas 2016). Hence, this study seeks to derive evidence on the continued use of eponyms in anatomy in the printed word (i.e., in anatomical sciences journals). As in any field, editors have a wide purview and insight into the innerworkings of publishing and consensus around field-specific terminology. Editors also have oversight and control of what is accepted into the journals they edit. Investigating the perceptions of editors on the challenges of eponym use, could thus yield insights and provide a way forward in the terminology debate that has been ongoing for roughly the last century (His 1895; Organ 1991; Fargen and Hoh 2014; FIPAT 2019; de Carvalho et al. 2022). In addition, the study sought to investigate whether the use of eponyms is ethical when considering the infamous history of some of the individuals after whom terms are named, whether eponyms fit into the concepts of diversity and inclusivity and quality of education.

Thus, the perspectives of journal editors on the ethics of continued eponym use in anatomical terminology and publishing was investigated.

## Materials and methods

Ethics clearance to undertake the study was obtained from the Human Research Ethics Committee (HREC) (Medical) of the University of the Witwatersrand (HREC no: M230505 M230526-0018).

This qualitative study made use of an online self-administered questionnaire (Supplementary Information 1) directed to the Editor-in-Chief or Senior Editor (referred to collectively as editors hereafter) of all international anatomy journals currently part of the Federative International Committee on Scientific Publication (FICSP) of the International Federation of Associations of Anatomists (IFAA). The sample included a total of 22 editors, each from their respective anatomy journals. The editors were contacted via email and were informed of the study and its purpose through a participant information sheet (Supplementary Information 2). Informed consent to participate in the study was requested.

Prior to distribution, the questionnaire was content validated by six senior local and international anatomists. Feedback from validators was incorporated into the questionnaire. A content validity ratio (CVR) (Lawshe 1975; Ayre and Scally 2014) was calculated for each question with multiple selection options in MS Excel. All questions with a CVR of 0.33 or lower were amended for clarity and relevance to the study aim (Supplementary Information 3: Table A1).

Test–retest was also conducted to confirm both internal consistency and replicability of the questionnaire. Internal consistency, on ten respondents was assessed by a Cronbach alpha. In addition, McNemar chi-squared tests were run with binary responses (e.g. yes/no), Mantel–Haenszel chi-squared tests were run with ternary or more nominal responses (e.g. yes/no/not sure), and an intraclass correlation (average measures, random raters) was run with ordinal responses (e.g. regularly/often/sometimes/rarely/never). All statistical analyses were conducted on R Studio (v. 4.3.2).

The survey included questions on demographics, including the age group that editors belonged to, countries in which they had worked as clinicians/academics, home language, the degrees held by each editor, years in clinical practice, years as editor, and numbers of journal edited. In addition, participants were asked their perspectives on the ethics and relevance of eponymous terminology usage in anatomy and in medicine. Open-ended questions were included. The final questionnaire (Supplemental Material 1) was distributed electronically via REDCap (Research Electronic Data Capture System). One reminder was sent approximately a week after the proposed deadline.

Due to the small number of respondents and the highly qualitative nature of the questions, analysis of responses was limited to frequencies of answers and open-ended responses were considered by combining response information into broader common themes where possible, particularly as some respondents provided lengthy responses that could not be easily related without extracting key conceptual information from them. As such, the open-ended questions were presented based on these broader themes identified within each set of responses. When no responses were provided in open-ended questions, these were not reported throughout the results; however, the overall number of respondents was still included in the totals (denominators) for each question.

## Results

### Questionnaire reliability

The overall internal consistency of the questionnaire was high (Cronbach-alpha = 0.834; bootstrapped 95% confidence interval = 0.583–0.903) but not too high (> 0.95) to indicate redundancy in the questionnaire design (Tavakol and Dennick 2011) (Supplementary Information 3: Table A2). The only question with a low reliability value was the frequency of encountering eponymous terms in the editorial office. This question requires memorization of details that might not be readily available, hence prone to inconsistent responses. However, this was considered an important question to view the usage of eponymous terms in anatomical publishing and was thus retained.

### Biographical overview of respondents

Of the 22 editors of anatomy journals, 16 (72.7%) responded to the questionnaire. Good regional representation of editors across the six continents was obtained. Editors had operated mainly out of Europe (*n* = 11, 68.8) or the USA (*n* = 7, 43.8%) for part of their careers (Table 1). None of the editors who responded were younger than 40 years of age, with half the editors being between 51 and 60 years of age (*n* = 8, 50%), or 61 and over (*n* = 5, 31.2%). Most editors had between one and 45 years (median of 10.5 years) in clinical practice, while five respondents did not have clinical experience. The majority (*n* = 9, 56.2%) of the respondents had 6 or more years of experience in their current roles as Editors-in-Chief or Senior Editors of an anatomical journal. None of the respondents had less than a year of experience in this position. Many editors have acted as editors for only one journal (*n* = 7, 43.8%).

**Table 1:** Countries of employment as anatomists and/or clinicians, for at least part of the Editors’ career

Continent of employment	Number of responses
Africa	3
Asia	4
Europe	11
Oceania	1
North America	7
South America	1
Total	27

As some editors were employed in multiple countries/regions, the total is greater than the individual number of respondents

### Relevance of eponymous terminology in anatomy

**Fig. 1:**
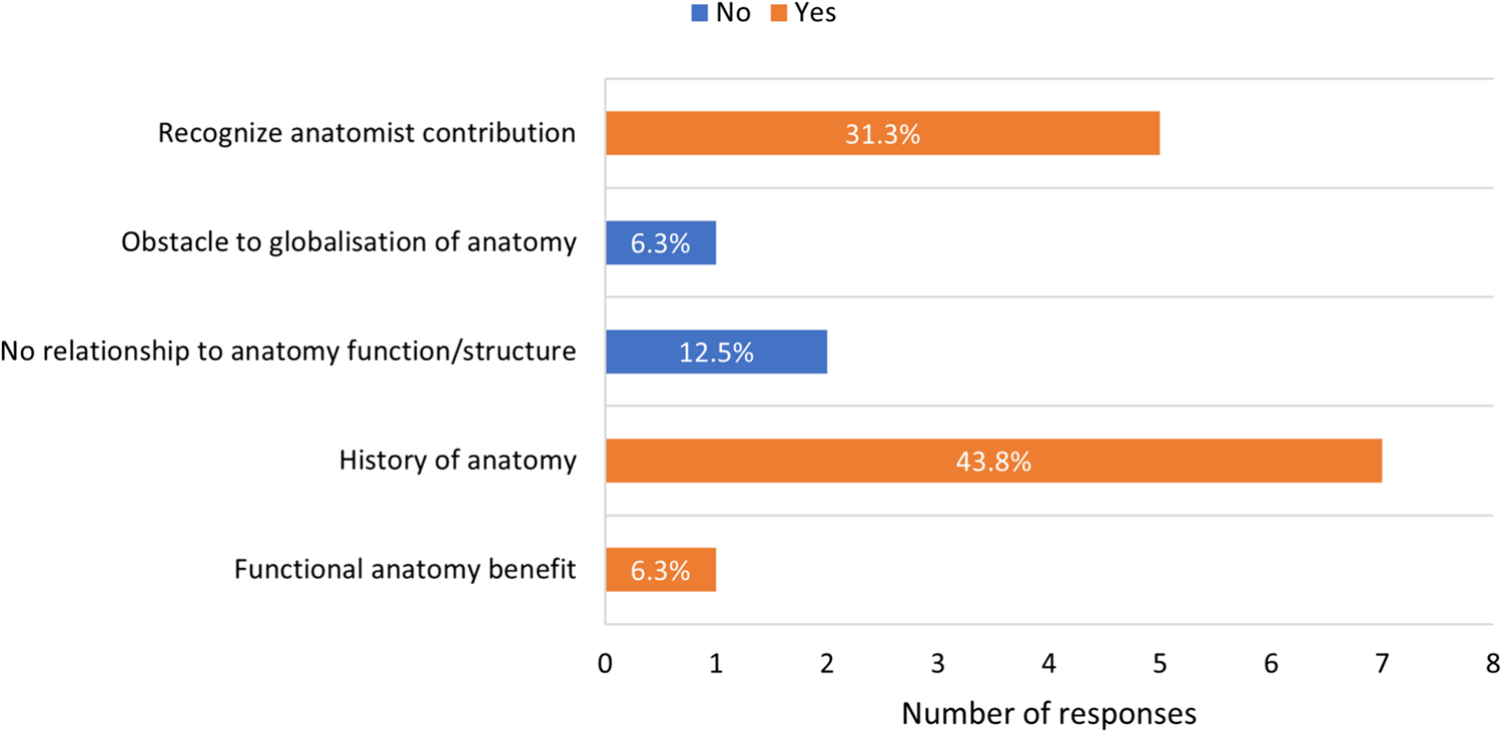
Reasons provided in support of or against the importance of eponyms. Results from thematic analysis are displayed in percentages of responses. Both respondents who considered eponyms as historically important and those that did not, were considered in the thematic analysis from question A, Table 2

The majority of responding editors agreed that eponymous terminology plays a vital role in the history of anatomy as a discipline (*n* = 13, 81.3%) (Table 2, question A). Reasons provided by these 13 editors were that eponymous terms function in exploring the history of anatomy and medicine (*n* = 7, 43.8%), or that they are an important way to recognize the contribution of anatomists in the discipline (*n* = 5, 31.3%) (Fig. 1). Negative responses included eponymous terms being unrelated to the anatomical structure and function of the “item” which they describe, and that they functioned as an obstacle to the globalization of anatomy (*n* = 3, 18.7%) (Fig. 1).

**Table 2:** Overview of eponymous terms in anatomy

	Question	Yes		No	
A	Do you consider eponyms an important aspect of the history of anatomy?	13	81.3%	3	18.7%
B	Were you aware that since the oldest edition of the *Nomina Anatomica* (His 1895) the use of eponymous terms was censured?	9	56.2%	7	43.8%
C	Are you aware that despite this, new eponymous terms have been coined in anatomy since 1895, and in some medical fields (e.g. Neurology) are still growing?	14	87.5%	2	12.5%
D	In your opinion, are there valid reasons why eponymous terms should not be used in anatomy training?	10	62.5%	6	37.5%
E	In your own opinion, are there valid reasons for reintroduction of eponymous terms in anatomical training?	9	56.2%	7	43.8%

Nine (56.2%) of the editors were aware that since the first *Nomina Anatomica* (His 1895), eponymous terms were censured in favor of single standard anatomical terms (Table 2, question B). When asked what they believe to be the reasons for the continued use of eponyms, the most common reason provided was the intransigence of clinicians and anatomists (*n* = 7, 43.8%). Other reasons cited were the historical relevance and recognition of anatomists’ contributions (*n* = 4, 21.0%), ease of use in comparison to the lengthy anatomical terms (n = 3, 18.8%), and a lack of integration between anatomy and clinical practices (*n* = 2, 12.5%).

Ten (62.5%) of the responding editors believed that there are valid reasons for the discontinuation of eponymous terminology in anatomy (Table 2, question D). Some of the reasons provided by the ten editors included that eponyms inhibited communication (*n* = 4, 40%) and that they are not meaningful to anatomists and clinicians (outside of highly specialized disciplines) (*n* = 2, 20%). Some respondents were not opposed to concurrent use (2/10, 20%), and some individuals were only in support of eponyms that contribute to understanding (e.g. Achilles tendon through reference to the mythological Achilles heel) (1/10, 10%). Among the editors that did not support the discontinuation of eponyms (37.5%) (Table 2, question D), half saw no valid reason for their withdrawal (3/6, 50%).

### Eponymous terminology in student and peer communication

Nine (56.3%) editors thought that eponyms should be reintroduced into anatomical training (Table 2, question E) but there was no consensus on the reasons provided. Reasons included historical relevance (2/9, 22.2%) and tradition (2/9, 22.2%), better clinician to student communication, out of necessity due to the literature saturated with eponyms, non-historical eponyms being beneficial, and that eponyms should be used concurrently with standard terminology. An editor who was against the reintroduction of eponyms, felt that they were limited in their usefulness to historical, humanistic and bioethical discussions.

**Fig. 2:**
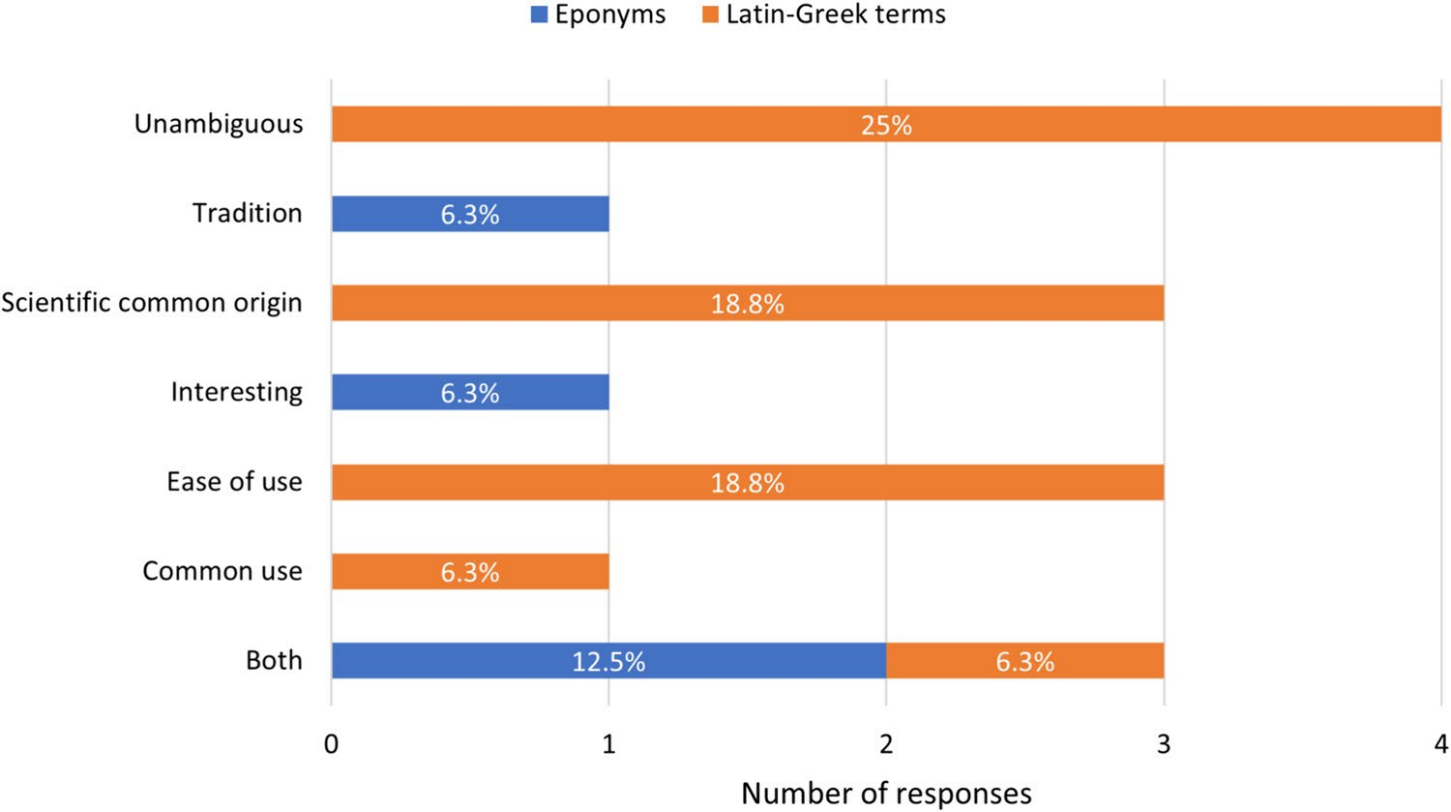
Preference of terminology usage in peer communication. Blue bars represent respondents who preferred eponymous terms and orange bars represent respondents who preferred Latin- and Greek-derived terms, with the associated justifications provided

**Fig. 3:**
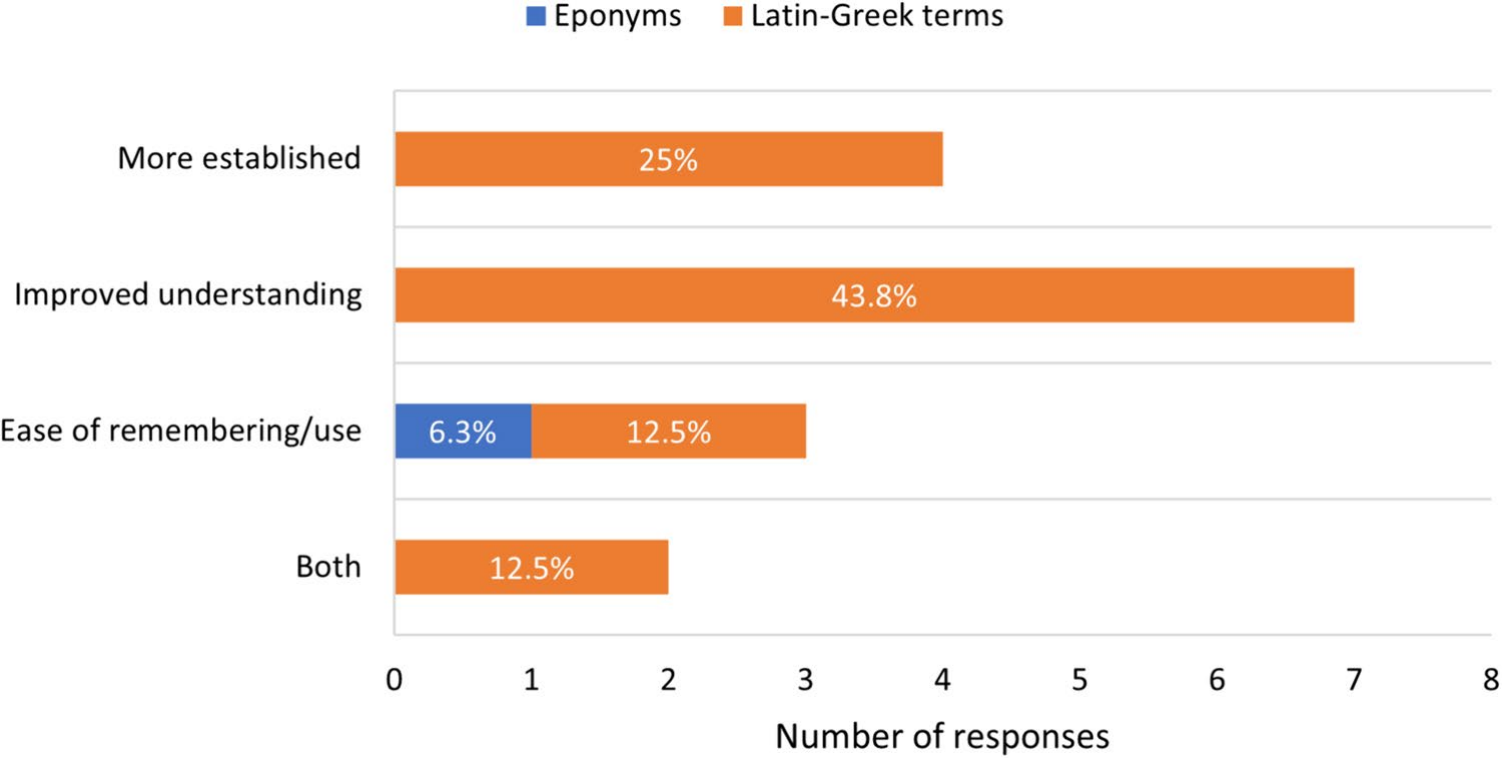
Preference of terminology usage in student communication. Blue bars represent respondents who preferred eponymous terms and orange bars represent respondents who preferred Latin- and Greek-derived terms, with the associated justifications provided

The majority of editors agreed that Latin- and Greek-derived anatomical terms were more useful both in communication with peers (*n* = 12, 75%) (Fig. 2) and moreso with students (*n* = 15, 93.8%) (Fig. 3). The most reported reason for Latin- and Greek-derived terms being preferred was unambiguous communication between peers (*n* = 4, 25%) and improved understanding for students (*n* = 7, 43.8%) (Figs. 2 and 3).

### Eponymous terminology attribution, inclusivity, infamy, and ethics

**Fig. 4:**
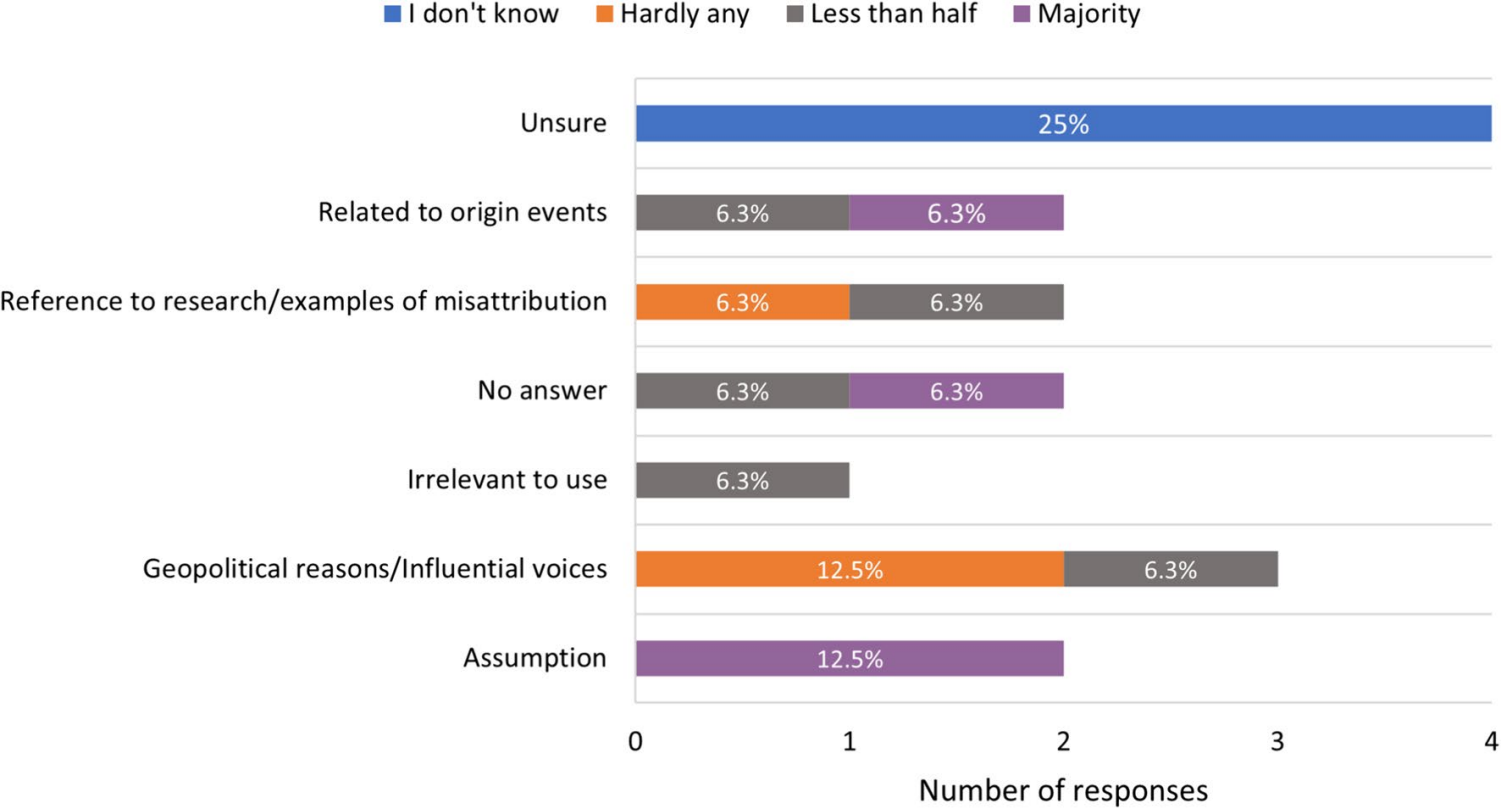
Views on correct attribution of eponymous terms. Blue bars represent respondents who did not know the proportion of correctly attributed eponyms, orange bars represent respondents who thought hardly any eponyms were correctly attributed, grey bars represent respondents who thought less than half of eponyms were correctly attributed and purple bars represent respondents who thought that the majority of eponyms are correctly attributed, with associated reasons for their views

Although there was no agreement on the proportion of eponyms that correctly acknowledge the discoverer, all editors agreed that most eponyms are associated with male individuals. Only three (18.8%) respondents were able to identify eponyms associated with a female individual, for example Nitabuch’s membrane, and in medical eponyms Sister Mary Joseph nodule and Frey syndrome. Two (12.5%) editors identified an eponym attributed to a Black, Indigenous, or person of colour (Aschoff-Tawara node) (Table 3, questions A and B). Some (*n* = 4, 25%) editors were unsure of the correct attribution of eponyms to their discoverer. Many (*n* = 8, 50%) editors believed that only less than half (*n* = 5, 31.3%) or hardly any (*n* = 3, 18.8%) of the eponyms correctly acknowledge the discoverer, and primarily saw eponyms as associated with the most influential voices and not necessarily the discoverer (Fig. 4).

**Table 3:** Eponymous terms in relation to historical injustices, infamy, and mythology

Question		Yes		No	
A	Are you aware of any eponyms related to a person of female gender, at the exclusion of the HeLa cells?	3	18.8%	13	81.2%
B	Have you encountered an eponym derived from an individual of Black, Indigenous, or People of Colour in anatomy, at the exclusion of the HeLa cells?	2	12.5%	14	87.5%
C	Do you believe that the use of the term “Clara cell” is appropriate/should be continued, despite its infamous history?	7	43.8%	9	56.2%
D	Do you believe that the use of the term “Bundle of His” is appropriate/should be continued, despite its infamous history?	10	62.5%	6	37.5%
E	Do you believe mythological eponyms, such as “Achilles tendon”, are more acceptable than historical ones?	6	37.5%	10	32.5%

“Clara cell” was found to be an inappropriate eponym by nine respondents (56.3%) (Table 3, question C) with six of the nine referring to the infamous historical relationship of Max Clara with the Nazi party. Seven editors (43.8%) supported the reference to Clara, suggesting that the context of the injustice should be clearly explained when used. However, the majority (*n* = 10, 62.5%) of editors did not object to the use of “Bundle of His” (Table 3, question D). Respondents identified that context should be explained from a historical perspective when these terms are used. Single respondents suggested that all eponyms should be removed irrespective of history, and that “Clara” in Latin-based languages can be etymologically confused with the definition of clear/pale. Those individual editors in support of the use of these infamous eponyms, responded that these terms are easy to use and remember, that they remain historically relevant, that other similarly infamous eponyms are widely used as well, and that terms should be used free of political connotations. Two editors (2/10, 20%) were also unaware of the infamy of these terms and hence were not opposed to their use. Most of those editors (*n* = 6, 37.5%) who specifically disagreed with the use of “Bundle of His”, suggested that His did not deserve this eponym (4/6, 66.7%).

The majority of editors (*n* = 10, 62.5%) also did not consider mythological eponyms more acceptable than historical ones (Table 3, question E). The respondents in support of mythological eponyms suggested that the well-known mythology helps to convey the anatomical structure and function (4/6, 66.7%).

Most editors (*n* = 10, 62.5%) were not in support of introducing new eponyms to reflect the emerging diversity of anatomists, or to reinstate eponyms in order to allow acknowledgment of inclusivity of minority group in anatomy and medicine. In opposition to the introduction of new eponyms to showcase diversity was that eponyms should only be attributed on merit and not just for the sake of diversity (4/10, 40%), and that the eponyms in existence are already a direct reflection of the history of the field with its lack of diversity. Others suggested that standard terminology is preferred, that no new eponyms should be introduced, but if they were, then being inclusive is important. Those who opposed the reintroduction of eponyms on the basis of inclusivity stated their reasons as being related to the risk of inaccuracy and potential for public backlash. Among the reasons provided in support of new eponyms for inclusivity was that this may help eliminate the perceived gender and population biases of the past (2/6, 33.4%) but were skeptical of whether they would be used widely enough to be successful (2/6, 33.4%). Overall, reinstating eponyms was seen as irrelevant to inclusivity or addressing injustices (3/10, 30%), but rather promoting historical injustices (5/10, 50%) and adding to cognitive load (2/10, 20%). Although many editors did not see the removal of eponyms as a form of decolonizing the curriculum (*n* = 7, 43.8%), a common theme among the respondents was that removal of eponyms is not effective, as other greater concerns around decolonization exist.

Eight (50%) of the responding editors saw the use of eponyms as ethical, while others (*n* = 5, 31.3%) were unsure. The latter group suggested this was a complex question that could relate to several aspects of ethics. According to the unsure respondents, the ethics of eponym use could relate to quality of education, historical injustices, or inclusivity. Among the editors that saw continued use of eponyms as unethical (*n* = 3, 18.8%), the common theme was to disavow the injustice of many eponyms (3/3, 100%), both in relation to historical injustice and marginalization.

Responses against reinstating eponymous terms (*n* = 10, 62.5%) often coincided (7/10, 70%) with those responses not in support of the attribution of new eponyms to themselves (*n* = 11, 68.8%). Reasons for not wanting eponyms attributed to themselves included a preference for the functional terminology over a new eponymous term (4/11, 36.4%), the unlikelihood of discovering a new structure and the history of anatomy already being too established (2/11, 18.2%), and the practice being narcissistic (2/11, 18.2%). One editor was provided with the opportunity to establish a new eponym and rejected it for the latter reason.

### Eponymous terminology in anatomy publications

**Fig. 5:**
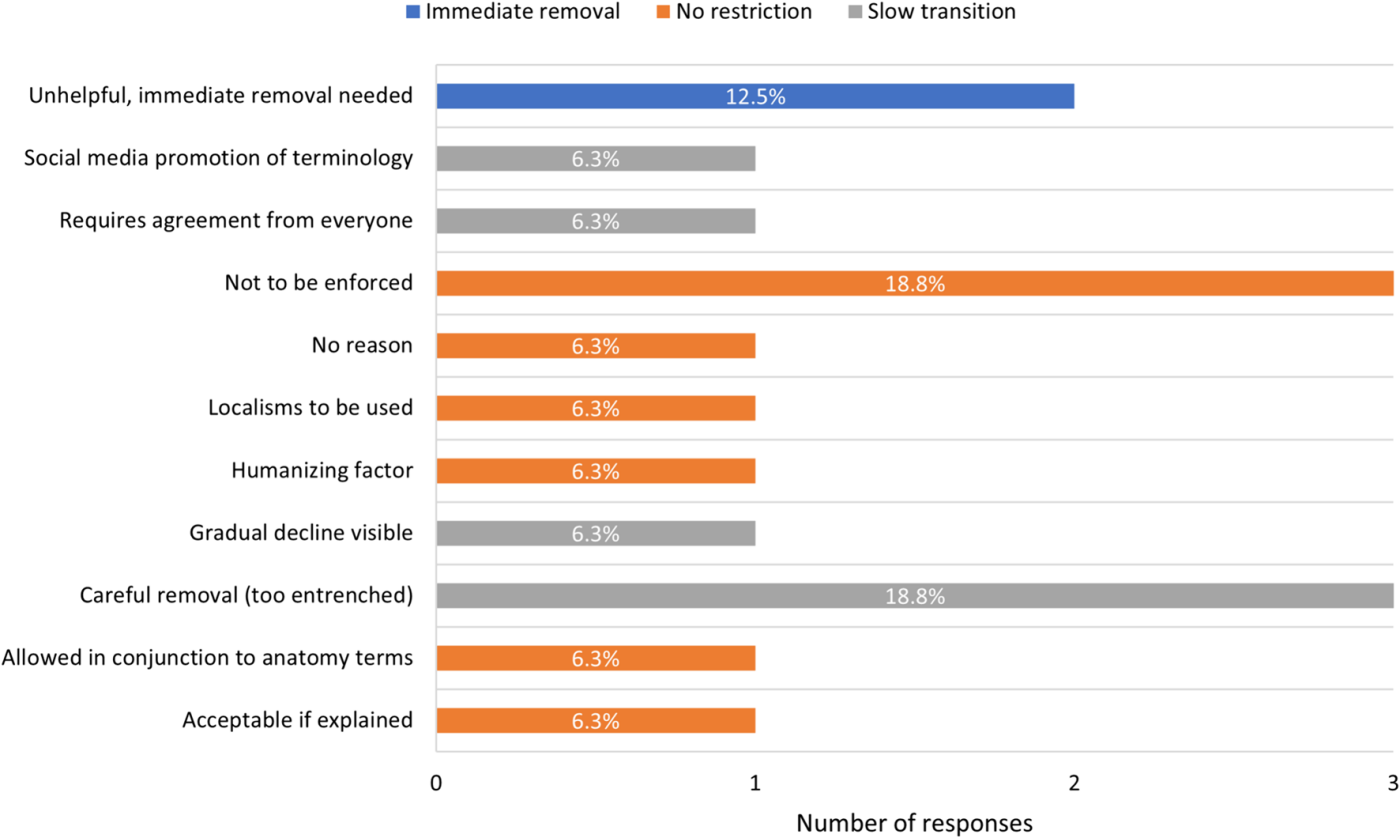
Recommended future of eponymous terms. Blue bars represent respondents who were in support of immediate removal of eponyms, orange bars represent respondents who supported no restrictions for eponyms, and grey bars represent respondents who supported a slow transition away from eponyms, with reasons associated to their views

In their position as editor, the majority of respondents (*n* = 14, 87.5%) came across eponyms sometimes (7/16, 43.8%) and often (7/16, 43.8%) (Supplementary Information 3: Table A3). Most editors were aware of the recommendation in TA2 on eponym use (*n* = 12, 75%) (Table 4, question B). Half of the editors followed these recommendations in their editorial office (*n* = 8, 50%) (Table 4, question C), with most of these eight editors (6/8, 75%) also agreeing that other journals should have restrictions on eponym use in their publications (*n* = 8, 50%) (Table 4, question D). However, the majority still considered eponyms to be appropriate in published work (*n* = 11, 68.8%) (Table 4, question A), most of these editors aligning their views (10/11, 90.9%) with their journal’s policy of accepting eponyms in manuscripts (*n* = 11, 68.8%) (Table 4, question E). When respondents viewed other anatomical journals, most either thought that the majority of journals did not follow the TA2 with regard to eponyms (*n* = 9, 56.3%), or were unsure (*n* = 3, 18.8%). Many respondents did not want any form of restriction on the use of eponymous terminology in anatomy (*n* = 8, 50%) (Fig. 5). Of those who did want eponym usage to be restricted in the scientific literature (*n* = 8, 50%), six of the eight (75%) suggested a slow transition and only two out of eight suggested immediate removal (25%) (Fig. 5).

**Table 4:** Eponym use in anatomy journals

Question		Yes		No	
A	Do you think the use of eponyms is appropriate in anatomical published work (textbooks, research papers etc.)?	11	68.8%	5	31.2%
B	Are you aware whether the *Terminologia Anatomica* 2 (TA2) has restrictions on the use of eponymous terminology?	12	75.0%	4	25.0%
C	In your role as editor, do you follow the TA2, with regard to the exclusion of eponyms, in your journal?	8	50.0%	8	50.0%
D	Should journals have restrictions on the use of eponyms in their publications?	8	50.0%	8	50.0%
E	Does the journal of which you are Editor-in-Chief/Senior Editor accept eponym usage in manuscripts?	11	68.8%	5	31.2%

When considering structured options in the survey, the continued use of eponyms in anatomical publications was primarily supported by editors on the grounds of historical significance (*n* = 12, 75%) and honoring scientists and physicians of great achievement (*n* = 10, 62.5%) (Table 5). Those who were against the continued use of eponyms in publications, cited amongst others the same eponyms being attached to different structures (*n* = 11, 68.8%), the concurrent use of multiple eponymous terms for the same structures (*n* = 9, 56.3%) and their controversial origin or associated history (*n* = 9, 56.3%), and their obfuscation of anatomical etymology (*n* = 9, 56.3%) (Table 6).

**Table 5:** Reasons for the use of eponyms in anatomical publications

Which of the following do you consider valid reasons for the use of eponymous terminology in anatomical publishing?	Agree	
Historical significance (legacy/tradition)	12/16	75.0%
Ability to simplify complex terminology	6/16	37.5%
Ability to prevent misunderstanding of terminology	4/16	25.0%
Addition of “character to the science	3/16	18.8%
Honoring scientists/physicians of great achievement	10/16	62.5%
Lending a philosophical/intangible aspect of anatomy/medicine	5/16	31.3%
Responsibility to acknowledge past anatomists	9/16	56.3%
Reminder of the humanity behind the science	8/16	50.0%
Lack of corresponding anatomical term for an eponym in *Terminologia Anatomica*	3/16	18.8%
Easy to remember, striking term	7/16	43.8%
None of the above	1/16	6.2%

**Table 6:** Reasons against the continued use of eponyms in anatomical publishing

Which of the following do you think are valid reasons for the continued non-use of eponymous terminology in anatomical publishing?	Agree	
Controversial origin or history	9/16	56.3%
Moral opposition to celebrating individual scientists/physicians	4/16	25.0%
Lacking scientific accuracy	8/16	50.0%
Lacking historic accuracy	6/16	37.5%
Lacking in diversity	4/16	25.0%
Lacking inclusivity	4/16	25.0%
Misappropriation of original discovery	6/16	37.5%
Obfuscating anatomical etymology	9/16	56.3%
Exclusionary nature (demographics and nationalities)	3/16	18.8%
Lack universality (not easily usable in multiple languages)	5/16	31.3%
Multiple eponymous terms used for the same structure	9/16	56.3%
Same eponym used for different structures	11/16	68.8%
Eponym inconsistency (e.g. spelling variations and possessive forms)	8/16	50.0%
Need for familiarity with the field	3/16	18.8%
None of the above	2/16	12.5%

Responding editors thought that regulation and recommendations around eponymous term usage should be governed by anatomical associations (*n* = 8, 50%), editors (*n* = 8, 50%), and reviewers (*n* = 6, 37.5%) (Table 7). Some (*n* = 4, 25%) editors suggested that multiple stakeholders should come together to provide guidance across all levels of eponymous terminology use.

**Table 7:** Stakeholders responsible on terminology recommendations regarding eponyms

Who in your opinion, should be responsible for determining whether the recommendations regarding eponyms of the *Nomina Anatomica* and the subsequent *Terminologia Anatomica*, and *Terminologia Anatomica* 2 are applied in anatomical publishing?	Agree	
Societies/associations	8/16	50.0%
Organizations	3/16	18.8%
Heads of departments of anatomy	5/16	31.3%
Universities	2/16	12.5%
Journal publishers	5/16	31.3%
Book publishers	4/16	25.0%
Editors	8/16	50.0%
Reviewers	6/16	37.5%
Scientists	5/16	31.3%
Physicians	3/16	18.8%
Clinicians	2/16	12.5%
Educators	4/16	25.0%
Other	2/16	12.5%
None of the above	2/16	12.5%

## Discussion

Although the use of anatomical eponyms is controversial (Organ 1991; Duque-Parra et al. 2006; Whitworth 2007; Strous and Edelman 2007; Whitworth and Matterson 2007; Jana et al. 2009; Gest 2014; Olry 2014a, b; Duque-Parra et al. 2018; McNulty et al. 2021; de Carvalho et al. 2022), eponyms were considered to be historically important by most editors. Despite the value placed on history, most editors surveyed preferred Latin- and Greek-derived terms over eponyms when communicating with both peers and especially students. The role of eponyms in historical recognition of anatomists was consistently touted as one of the reasons in support of eponyms, even when they were the product of infamous practices. No consensus was found regarding inclusivity, curriculum decolonization, or whether continued eponymous term use is ethical in the limited context provided. Editors in support of the continued use of eponyms did not consider attribution of terms for inclusivity a good strategy, while those in support of strategies for inclusivity still did not consider introduction of new eponymous terms beneficial.

There was limited consensus on views around the future of eponyms in anatomy, even when editors were in support of eponym use from a philosophical perspective. When specifically asked about anatomical communication, the overwhelming majority of editors preferred Latin- and Greek-derived terms over eponyms. The opinions of the editors mirrored the arguments in the literature towards the benefits of Latin- and Greek-derived terminology in their unambiguity, functional benefit and ease of use, universality in the language of science, and their established presence (Whitworth and Matterson 2007; Werneck and Batigália 2011; Olry 2014a; Wisner et al. 2022). Allied health undergraduate students were better versed in understanding and identifying anatomical structures when descriptive/functional terms were used in comparison with eponyms (Wisner et al. 2022). Some respondents in the current study suggested that eponyms are in fact not meaningful to anatomy students and inhibitory to communication, with one respondent stating:

*“[T]he use of medical eponyms, as in other areas, is often random, inconsistent, idiosyncratic, confusing, and even misleading.” (Respondent 11)*

Tradition, appropriate recognition of scientific achievement and historical importance are the primary counterarguments in support of eponymous term use, both in this study and in the literature (Whitworth 2007; Whitworth and Matterson 2007; Popkin et al. 2013; Fargen and Hoh 2014; Olry 2014b). Suggesting that eponymous terms are the traditional approach to nomenclature in anatomy (and medicine) is historically incorrect. Latin- and Greek-derived terms have been at the core of anatomy (and medicine) for a significantly longer period of time (Werneck and Batigália 2011) than other more modern languages that most eponyms are associated with:

*“Much of the textbooks use Latin-Greek based terms. For teaching this is most important to have similar names in different textbooks. Latin-Greek nomenclature has been used for over 2000 years. English nomenclature is used maybe last 50 years?” (Respondent 2)*

From an educational perspective, a descriptive and functional approach in anatomical teaching allows for deep learning of anatomy (Barger 2019). Yet eponyms, which enforce memorization over understanding (Barger 2019) continue to be used in practice, teaching and publications (Werneck and Batigália 2011), particularly by clinicians and surgeons (Strzelec et al. 2017). The intransigence of anatomists and clinicians was considered as one of the primary reasons for the continued use of eponyms by the majority of responding editors in this study. Clinicians, despite not being aware of the history behind eponyms, were indeed found to use eponyms with peers and believed that eponyms should be included in training (Wisner et al. 2023). Despite the eponyms’ ambiguity, most responding editors still supported their use in publications from a historical point of view. Irrespective of the stance of anatomists, many of the responding editors agreed that eponyms can contribute to a more in-depth understanding of history and ethics of the discipline, for example:

*“Eponyms are a window into the history of anatomy. They provide an opportunity to examine the ethical and unethical histories of our discipline and the development of bioethics.” (Respondent 12)*

The role of bioethics in clinical practice is underplayed by a lot of medical training institutions (Wynia et al. 2015), as often preference is given to clinical over bioethics training (Ngan and Sim 2021). By integrating eponyms in anatomy training to emphasize the historical injustices and ethical transgressions, bioethics training can be initiated in the pre-clinical years (Barger 2019; Organ and Mussell 2021) and continued into the clinical years. Awareness of the history behind eponyms, influenced whether clinicians would be willing to use eponyms (Wisner et al. 2023). Paving a way forward by introducing the history of eponymous terms in anatomy and medical training would not only be beneficial to early bioethics training but also to transition away from, at the very least, those with questionable or outright infamous historical connotations.

Recognition deservedness of eponyms is debatable. Although 25% of editors thought that the majority of eponyms are correctly attributed, the reality is that multiple studies have identified the misattribution of eponyms as widespread (Aresti and Ramachandran 2012; Olry 2014a; McNulty et al. 2021; Andrew et al. 2023). Historical misattribution of eponyms is pervasive, with many associated with the most influential voices of the time and the discipline (Whitworth and Matterson 2007; Koshlakov et al. 2019). This reinforces the argument that eponyms are attributed in a lottery-like fashion (Koshlakov et al. 2019) to anatomists/physicians who published in journals and/or languages more broadly accessible for the time (Whitworth and Matterson 2007), when not driven by fame, luck and political agendas (Whitworth and Matterson 2007; Koshlakov et al. 2019), or the compulsion for scientific recognition (Guptha Munugoor Baskaran et al. 2012). Despite this, certain editors considered honoring discoverers with an eponym as a legitimate, universal expectation:

*“Everyone wants to live forever and so do scientists” (Respondent 15)*.

However, other editors saw the desire for recognition as a narcissistic and shameful practice:

*“That’s very narcissistic. When someone discovers a new species, they’re not allowed to name it after themselves. The same principle should apply to any discovery, including Anatomical structures” (Respondent 3)*.

Eponyms being considered as an egotistical practice is not a new perspective (Squires 1989). In using eponyms there is also always the risk of falling prey to the ‘Matthew effect’, whereby eponyms or discoveries are ascribed to the head of a laboratory instead of the actual discoverer (Olry and Haines 2012). Historically, medical students saw this practice as a way of exhibiting appreciation to their teachers whom they venerated (e.g. Gasser’s ganglion) (Olry 2014b), but in practice, it stripped the students of recognition for their achievements.

The reinforcement of misattributed eponyms or lack of attribution of eponyms particularly disadvantages women scientists and physicians. This was evident when all editors agreed that eponyms attributed to men occur significantly more often than those attributed to women. A recent review found that only 4% of all medical eponyms investigated were attributed to women (Stuart-Smith et al. 2022). While this is argued as an accurate representation of the ‘golden era of eponymizing’ in anatomy and medicine which was historically predominantly white male dominated (Stuart-Smith et al. 2022), the lack of women in eponymous terminology persists as a reminder of the historic lack of inclusivity in the discipline. This was reinforced by the current study in which the overwhelming majority of responding editors were not able to name eponyms associated with women or people of colour. Some editors commented that they had not considered the lack of diversity in eponymous terms before, for example:

*“To be honest, I have never thought about this before, because this point has not played a role in [COUNTRY REDACTED FOR ANONIMITY] so far and we have not had any black people in our anatomical society so far and there are only very few among our students, so this has not been an issue for us so far, but diversity is extremely important.” (Respondent 9)*.

As the majority of published research in anatomy is centralized around Western nations, or reliant on white male anatomists’ illustrations and descriptions (Stuart-Smith et al. 2022), this paucity of awareness of the lack of diversity and inclusivity might be more pervasive than expected, and awareness of controversies around eponymous terms should be raised. Certain anatomists have taken it upon themselves to do just that, primarily through social media and university news outreach platforms (e.g. Ferguson 2022; ANATOMYINCLAY 2023).

Use of inappropriate or infamous eponyms [Clara cell, named after Max Clara who was associated with the Nazi party and used tissue from executed prisoners (Winkelmann and Noack 2010); and Bundle of His, named after Wilhelm His Jr who was an advocate for eugenics in relation to mental illness and ‘racially compromised’ individuals (Strous and Edelman 2007)] was not a primary concern for many editors and some were even uncertain of the infamy attributed to them. The fact that these were considered more appropriate than mythological eponyms was interesting and likely suggestive of the historical importance attributed by many editors to eponyms, over the potential functional benefit of certain mythological ones (e.g. Achilles tendon, Ammon’s horn, Atlas vertebra etc.). As the historical importance of eponyms is considered highly relevant to their continued use, their infamy should be highlighted, and the context of the discovery discussed when these eponyms are introduced in the classroom.

A hypothetical proposition in the current study was to reintroduce eponyms to include demographically diverse anatomists that are currently emerging as scientists and clinicians. This was met with resistance by multiple editors as they saw the risk that these new eponyms would not become prominent enough in anatomy or medicine to achieve inclusivity. It was recommended that historical curiosity be directed to the real stories behind the scientific discoveries and credit individual contributions appropriately:

*“…Rather than employing eponyms, we ought to leverage our curiosity about medical history to present impartial and accurate narratives of scientific breakthroughs and to analyse individual contributions.” (Respondent 11).*

Perspectives around whether inclusivity is considered a part of ethics are varied and often very personal. However, equity and inclusivity are embodied in ethical practice through the Belmont report Principle of Justice in subject selection in the context of equal protection and equal opportunity (Nagai et al. 2022). The question on the ethics around continued use of eponyms did not have consensus with only half of the responding editors finding eponyms unethical, primarily from an injustice perspective. This could likely be a result of the breadth of the survey question allowing for multiple interpretations and uncertainty among respondents:

*“This question can be related to ethics in so many ways: ethical to our students’ learning? ethical because of past injustices? This is a complicated question.” (Respondent 12)*.

In publications, surveyed editors encountered eponyms regularly (often and sometimes) and only half applied the TA2 in relation to eponym use in their editorial office, despite most of the responding editors considering eponyms appropriate in published literature. This is in alignment with studies investigating eponym prevalence across medical literature (Werneck and Batigália 2011; Thomas 2016; Zheng and Gold 2020). Although a decrease in the variety of eponyms frequently used has been noted in the last thirty years (Thomas 2016), many fields still see rampant use of eponyms (Zheng and Gold 2020). As it seems that at least some eponyms are so entrenched that removal will be difficult to impossible, a recommendation to only make use of eponyms with no dubious historical connotations and ensure that they are followed by the descriptive anatomical term (Latin/Greek-derived) may be a feasible middle ground in the eponym debate in publication. This recommendation is particularly relevant as half of the surveyed editors were not in support of restricting eponym use in journals, suggesting that a middle ground must be found in order to achieve some form of resolution to the eponym debate. Whichever stance editors chose, their purview and control of terminology in anatomy can assist with shaping the future of anatomical terminology. By means of editorial oversight, editors have the authority and influence to ensure a particular use, and enforce or reject recommendations, as was previously done with regard to acknowledging body donors in anatomical studies (Iwanaga et al. 2021). Hence, their responsibility in the eponym debate may be generally understated, particularly as half of the responding editors thought it is their responsibility to do so.

## Limitations

As this study had a small sample size due to the purposive sampling, statistical analyses did not yield impactful results and data presented is strictly based on frequencies of responses and qualitative assessment (thematic analysis) of open-ended responses. Considering the varied responses, perspectives and limited consensus in most survey questions, conclusive interpretation of the results was difficult. Particularly, the question around ethics of eponym use was broad and the majority of respondents were not able to pinpoint ethical concerns in such a broad context. More directed questions or structured interviews concerning the ethics and inclusivity of eponym use may have allowed a better understanding of the various aspects of ethical involvement in the contexts of the quality of anatomy teaching, historical injustices, and the exclusion of underrepresented groups. The purposive sampling of the study, although in clear alignment with its aim and representative of editors of anatomy journals, also may have contributed to some level of selection bias, particularly in relation to age, gender, and nationality, which does not necessarily represent the current view of younger more diverse anatomists.

## Conclusion

The documented intransigence of anatomists and clinicians to move away from eponyms and the lack of consensus on eponym use from editors in the current study suggests that the debate on eponym use will persist for some time, particularly as half of the respondents were not willing to restrict eponym use in journal publications. While the majority of responding editors agreed that in communication, eponyms are not as useful as Latin- and Greek-derived anatomical terms, this level of consensus was lacking across most other posed questions. One could conclude that eponyms are perceived as being detrimental to student learning, yet beneficial to the history and ethics of anatomy and that the latter is a compelling reason for retaining their use in some form. However, more in-depth and widespread studies should be conducted to interrogate the use of eponyms from the perspectives of junior and senior anatomists, and clinicians, especially in relation to cognitive load and diversifying the discipline. In the long term, with more evidence, a combined effort from anatomical terminology committees and stakeholders in anatomical publishing across the globe must consider similar findings to thoroughly review the terminologies and develop a guiding policy around eponym use or non-use based on compromise of opinions, and with an open-minded and inclusive approach.

### Supplementary Information

Below is the link to the electronic supplementary material.

Supplementary file1 (PDF 75 KB)

Supplementary file2 (PDF 153 KB)

Supplementary file3 (PDF 144 KB)
