# Supplementary material for: Perceptions of journal editors on the use of eponyms in anatomical publishing: the need for compromise
Source: Anat Sci Int. 2024 Jul 17;99(4):441–53. doi: 10.1007/s12565-024-00789-z (PMC11303421; doi:10.1007/s12565-024-00789-z)
Supplement: Supplementary file 1 — Supplementary file1 (PDF 75 KB) [file 12565_2024_789_MOESM1_ESM.pdf]

Title: Perceptions of journal editors on the use of eponyms in anatomical publishing: the need for compromise
Journal name: Anatomical Sciences International
Authors: Nicholas Bacci*, Erin Hutchinson, Beverley Kramer and Brendon Kurt Billings,
*Corresponding author
Affiliation: School of Anatomical Sciences, Faculty of Health Sciences, University of the Witwatersrand, Johannesburg
Address: School of Anatomical Sciences, University of the Witwatersrand Medical School, Office 2B04, 2nd Floor, 7 York Road, Parktown, Johannesburg, South Africa, 2193
Email: nicholas.bacci@wits.ac.za

Supplementary Information 1: Self-Administered Questionnaire

Ethics and Eponyms Questionnaire

Please complete the survey below.

Thank you!

Instructions and Consent

Good day,

My name is Nicholas Bacci, and I am a lecturer in the School of Anatomical Sciences, University of the Witwatersrand, Johannesburg, South Africa. I would like to invite you to participate in a research study on the use of eponyms in anatomy. While the terminologies of the International Federation of Associations of Anatomists (IFAA) no longer recognise the use of eponyms as part of anatomical terminology, certain eponyms appear to persist, both in language and in print.

By definition, an eponymous term is a term that originates from the name of a person which is applied to an anatomical structure. The term is generally either the name of a real person (e.g., circle of Willis) or of a fictitious individual (e.g., Achilles tendon) (Gest, 2014).

The goal of this study is to determine the perceptions of Editors-in-Chief/Senior Editors of anatomical journals on continued eponym usage in anatomical teaching, research, and publishing. This study is being conducted among all the Editors-in-Chief/Senior Editors of the IFAA recognised anatomical journals throughout the globe. Your key insight into these matters will allow the greater scientific community to have a better understanding of the considerations of senior editors when interacting with anatomical research.

The study involves a confidential, anonymous, self-administered questionnaire requesting your personal perspectives and opinions regarding the ethics of the continued use of eponymous terms in anatomy. While we are targeting specific individuals in Editor-in-Chief/Senior Editor roles, your responses will not be tied to your person and no directly identifying personal details will be captured as part of the survey. Your participation in this study is completely voluntary, and there will be no penalty or loss of any benefits if you do not want to participate. If you do decide to participate, no personal financial gain or academic benefit will be provided. No direct connections will be made between individual journals or individuals and their responses. These questions and responses are intended to represent your substantial experience as an editor of a major anatomical journal.

Accepting these conditions and continuing to the questionnaire will be considered as your informed consent to participate in the study.

Thank you for your willingness to participate.

The research team,

Dr Nicholas Bacci, Professor Beverley Kramer, Dr Erin Hutchinson, Dr Brendon Billings.

Reference:

Gest, T. R. (2014) 'Anatomical nomenclature and the use of eponyms', Clinical Anatomy, 27(8), p. 1141. doi: 10.1002/ca.22407.

Do you agree to participate in the following study as described above and in the information sheet provided?

◯ Yes
◯ No

Demographic Information

Please state your age.	◯ Below 40 years<br>◯ 41 to 50 years<br>◯ 51-60 years<br>◯ 61 and over
Please list in which countries you have worked as an anatomist or a clinician. (Please separate each country with a comma).	__________________________________
What is your primary/home language?	__________________________________
List your undergraduate and postgraduate degrees and the years in which each were obtained (e.g., MBBCh, 1984; PhD, 1989 etc.).	__________________________________
Please state the number of years in which you have worked in a clinical context, if any (state "0" if none).	__________________________________
How many years have you been in your current post as an editor of an anatomical journal?	◯ less than 1 year<br>◯ 1 to 5 years<br>◯ 6 to 10 years<br>◯ more than 10 years
How many different anatomical journals have you edited?	__________________________________

Relevance

Definitions of terms used in the survey:

Eponym (Merriam-Webster Dictionary online: https://www.merriam-webster.com/dictionary): One for whom or which something is or is believed to be named (e.g., Achilles tendon, Band-aid). A name (as of a drug or a disease) based on or derived from an eponym (e.g., Down syndrome). Eponymous (Merriam-Webster Dictionary online: https://www.merriam-webster.com/dictionary): Of, relating to, or being the person or thing for whom or which something is named. Of, relating to, or being an eponym. Latin-Greek derived anatomical terms (ad hoc definition by study group) An anatomical term derived from a classical Latin and/or Greek origin with etymological descriptive and/or functional associations and supported by the Nomina Anatomica, Terminologia Anatomica, or Terminologia Anatomica 2 (e.g., sympathetic trunk/truncus sympathicus).

Do you consider eponyms an important aspect of the history of anatomy?

○ Yes
○ No

Please provide a reason for your answer.

____

Were you aware that since the oldest edition of the Nomina Anatomica (His, 1895), the use of eponymous terms was censured?

His, W. (1895) Nomina Anatomica. 1st edn. Leipzig, Germany: Veit & Comp. Available at: https://ia800507.us.archive.org/23/items/dieanatomischeno00hisw/dieanatomischeno00hisw.pdf.

○ Yes
○ No

Are you aware that despite this, new eponymous terms have been coined in anatomy since 1895, and in some medical fields (e.g., Neurology) are still growing?

○ Yes
○ No

List, in order of importance (from most important to least important), the reasons why you think eponyms continue to be used in clinical practice despite their condemnation by anatomical terminology committees.

____

In your own opinion, are there valid reasons why eponymous terminology should not be used in anatomy training?

○ Yes
○ No

Please elaborate.

____

In your own opinion, are there valid reasons for the reintroduction of eponymous terminology in anatomical training?

○ Yes
○ No

Please elaborate.	__________________________________
Which do you find more useful in peer communication: eponymous terms or Latin-Greek derived anatomical terms?	◯ Eponymous terms<br>◯ Latin-Greek derived anatomical terms
Please elaborate.	__________________________________
Which do you find more useful in student learning: eponymous terms or Latin-Greek derived anatomical terms?	◯ Eponymous terms<br>◯ Latin-Greek derived anatomical terms
Please elaborate.	__________________________________

Ethics, Diversity, and Inclusivity

Definitions (Merriam-Webster Dictionary online: https://www.merriam-webster.com/dictionary):

Inclusivity The quality or state of being inclusive: including everyone, especially: allowing and accommodating people who have historically been excluded (as because of their race, gender, sexuality, or ability). Diversity The condition of having or being composed of differing elements; variety, especially: the inclusion of people of different races, cultures, etc. in a group or organization.

What proportion of eponymous terms acknowledge correctly who contributed to their discovery?	◯ Majority<br>◯ Less than half<br>◯ Hardly any<br>◯ I don't know
Please elaborate.	__________________________________
Are most eponyms associated with male or female individuals?	◯ Male<br>◯ Female
Are you aware of any eponyms related to a person of female gender, at the exclusion of the HeLa cells?	◯ Yes<br>◯ No
If yes, please provide an example.	__________________________________
Have you encountered an eponym derived from an individual of Black, Indigenous, or People of Colour in anatomy, at the exclusion of the HeLa cells?	◯ Yes<br>◯ No
If yes, please provide an example.	__________________________________
As anatomy and the sciences are becoming more inclusive, do you think that the attribution of new eponyms should be introduced to showcase the emerging diversity across the newly included individuals of diverse backgrounds and genders?	◯ Yes<br>◯ No
Please elaborate.	__________________________________
If you discovered a novel structure, would you like to see your own name associated with it as a form of acknowledgement of your achievement?	◯ Yes<br>◯ No
Please provide reasons for your answer.	__________________________________

Do you believe that the removal of eponyms from anatomical terminology would be an effective step towards decolonising the health sciences curriculum?<br><br>Definition (Merriam-Webster Dictionary online: https://www.merriam-webster.com/dictionary):<br><br>Decolonise<br><br>To free from the dominating influence of a colonising power; especially: to identify, challenge, and revise or replace assumptions, ideas, values, and practices that reflect a coloniser's dominating influence and especially a Eurocentric dominating influence.	◯ Yes<br>◯ No<br>◯ Not sure
Please elaborate.	__________________________________
Do you believe that the use of the term "Clara cell" is appropriate/should be continued, despite its infamous history?	◯ Yes<br>◯ No
Please provide reasons for your answer.	__________________________________
Do you believe that the use of the term "Bundle of His" is appropriate/should be continued, despite its infamous association?	◯ Yes<br>◯ No
Please provide reasons for your answer.	__________________________________
Do you believe mythological eponyms, such as "Achilles tendon", are more acceptable than historical ones?	◯ Yes<br>◯ No
If yes, do you believe this type of eponym is appropriate? Why?	__________________________________
Should eponyms be fully reinstated in official terminology in order to acknowledge the increasing inclusivity and diversity of anatomical and medical fields, particularly in an attempt to address historical injustices?	◯ Yes<br>◯ No
If yes, how would you suggest that diversity is included in a reintroduction of eponyms?	__________________________________
If no, please elaborate.	__________________________________

Is it ethical to continue using eponymous terms in anatomy?	◯ Yes<br>◯ No<br>◯ Not sure
Please elaborate.	__________________________

Academic Publishing

How often do you encounter eponymous terminology in manuscripts received by your editorial office?

- ◯ Regularly
- ◯ Often
- ◯ Sometimes
- ◯ Rarely
- ◯ Never
- ◯ Not sure

Do you think the use of eponyms is appropriate in anatomical published work (textbooks, research papers etc.)?

- ◯ Yes
- ◯ No

Please elaborate.

____

Are you aware whether the Terminologia Anatomica 2 (TA2) has restrictions on the use of eponymous terminology?

- ◯ Yes
- ◯ No

In your role as an editor, do you follow the TA2, with regard to the exclusion of eponyms, in your journal?

- ◯ Yes
- ◯ No

Do you think the exclusion of eponyms as prescribed in TA2 is applied by most anatomical journals?

____

Which of the following do you consider valid reasons for the use of eponymous terminology in anatomical publishing? (Select all that apply)

- ☐ Historical significance (legacy/tradition)
- ☐ Ability to simplify complex terminology
- ☐ Ability to prevent misunderstanding of terminology
- ☐ Addition of "character" to the science
- ☐ Honoring scientists/physicians of great achievement
- ☐ Lending a philosophical/intangible aspect of anatomy/medicine
- ☐ Responsibility to acknowledge past anatomists
- ☐ Reminder of the humanity behind the science
- ☐ Lack of corresponding anatomical term for an eponym in Terminologia Anatomica
- ☐ Easy to remember, striking term
- ☐ Other (elaborate below)
- ☐ None of the above

If "Other" was selected, please elaborate.

____

Which of the following do you think are valid reasons for the continued non-use of eponymous terminology in anatomical publishing? (Select all that apply)	☐ Controversial origin or history<br>☐ Moral opposition to celebrating individual scientists/physicians<br>☐ Lacking scientific accuracy<br>☐ Lacking historic accuracy<br>☐ Lacking in diversity<br>☐ Lacking in inclusivity<br>☐ Misappropriation of original discovery<br>☐ Obfuscating anatomical etymology<br>☐ Exclusionary nature (demographics and nationalities)<br>☐ Lack of universality (not easily usable in multiple languages)<br>☐ Multiple eponymous terms used for the same structure<br>☐ Same eponym used for different structures<br>☐ Eponym inconsistency (e.g., spelling variations and possessive forms)<br>☐ Need for familiarity with the field<br>☐ Other (elaborate below)<br>☐ None of the above
If "Other" was selected, please elaborate.	__________________________________
Should journals have restrictions on the use of eponyms in their publications?	◯ Yes<br>◯ No
Please elaborate.	__________________________________
Does the journal of which you are Editor-in-Chief/Senior Editor accept eponym usage in manuscripts?	◯ Yes<br>◯ No
Who, in your opinion, should be responsible for determining whether the recommendations regarding eponyms of the Nomina Anatomica and the subsequent Terminologia Anatomica, and Terminologia Anatomica 2 are applied in anatomical publishing? (Select all that apply)	☐ Societies/associations<br>☐ Organisations<br>☐ Heads of Departments of Anatomy<br>☐ Universities<br>☐ Journal publishers<br>☐ Book publishers<br>☐ Editors<br>☐ Reviewers<br>☐ Scientists<br>☐ Physicians<br>☐ Clinicians<br>☐ Educators<br>☐ Other: elaborate below<br>☐ None of the above
If "Other" was selected, please elaborate.	__________________________________
Please elaborate on your choices.	__________________________________

If you believe that eponyms should be restricted in their use, should they be transitioned out of the scientific literature and practice, or be immediately removed from all literature and practical use?	◯ Slow transition<br>◯ Immediate removal<br>◯ No restriction
Please elaborate.	__________________________________
If transitioned slowly, what do you believe the best avenue for this would be?	__________________________________
If urgently removed, what do you believe the best avenue for this would be?	__________________________________
