# Supplementary material for: Perceptions of journal editors on the use of eponyms in anatomical publishing: the need for compromise
Source: Anat Sci Int. 2024 Jul 17;99(4):441–53. doi: 10.1007/s12565-024-00789-z (PMC11303421; doi:10.1007/s12565-024-00789-z)
Supplement: Supplementary file 2 — Supplementary file2 (PDF 153 KB) [file 12565_2024_789_MOESM2_ESM.pdf]

Title: Perceptions of journal editors on the use of eponyms in anatomical publishing: the need for compromise
Journal name: Anatomical Sciences International
Authors: Nicholas Bacci*, Erin Hutchinson, Beverley Kramer and Brendon Kurt Billings,
*Corresponding author
Affiliation: School of Anatomical Sciences, Faculty of Health Sciences, University of the Witwatersrand, Johannesburg
Address: School of Anatomical Sciences, University of the Witwatersrand Medical School, Office 2B04, 2nd Floor, 7 York Road, Parktown, Johannesburg, South Africa, 2193
Email: nicholas.bacci@wits.ac.za

<u>Supplementary Information 2: Participant Information Sheet</u>

PARTICIPANT INFORMATION SHEET

Ethics and Eponyms: Perceptions of journal editors on the continued use of eponyms in anatomical terminology and publishing

Good day,

My name is Nicholas Bacci, and I am a lecturer in the School of Anatomical Sciences, University of the Witwatersrand, Johannesburg, South Africa. I would like to invite you to participate in a research study on the use of eponyms in anatomy. While the terminologies of the International Federation of Associations of Anatomists (IFAA) no longer recognise the use of eponyms as part of the anatomical terminologies, certain eponyms appear to persist, both in language and in print.
By definition, an eponymous term is a term given to an anatomical structure that originates from the name of an individual. The term is either the name of a real person (e.g., circle of Willis) or of a fictitious individual (e.g., Achilles tendon) (Gest, 2014).

The goal of this study is to determine the perceptions of editors of anatomical journals on the continued eponym usage in anatomical teaching, research, and in publishing. This study is being conducted among all the senior editors of the IFAA recognised anatomical journals throughout the globe.
Your insight into these matters will allow the greater scientific community to have a better understanding of the considerations of senior editors when interacting with anatomical research. As in your role as editors you are the key individuals exposed to the most current and cutting-edge research in the field of anatomy.

The study involves a confidential, anonymous, online, self-administered questionnaire requesting your personal perspectives and opinions regarding the ethics of the continued use of eponymous terms in anatomy. The questionnaire should take no longer than 15 to 30 minutes. Your participation in this study is completely voluntary, and there will be no penalty or loss of any benefits if you do not want to participate. If you do decide to participate, no personal financial gain or academic benefit will be provided. No direct connections will be made between individual journals or individuals and their responses. These questions and responses are intended to represent your substantial experience as an editor of a major anatomical journal. As no directly identifying information will be collected your risk in participating in the study is minimal, if any.

Engaging with the online questionnaire implies that you have consented to take part in the study. Should you change your mind while completing the study you are welcome to stop answering the questionnaire and ask to withdraw your responses from inclusion in the study, at no penalty. Your specific data will then be destroyed and confirmation of this will be provided by the PI of the study unless you specifically consent to its retention. If you decide to participate and after the results are available, you are entitled to a free summary of these results, upon request. All data collected in the course of the study will be securely retained for five (5) years. Thereafter it will be destroyed accordingly.

Thank you for your willingness to participate.

The research team,
Dr Nicholas Bacci, Professor Beverley Kramer, Dr Erin Hutchinson, Dr Brendon Billings.

Reference:

Gest, T. R. (2014) ‘Anatomical nomenclature and the use of eponyms’, *Clinical Anatomy*, 27(8), p. 1141. doi: 10.1002/ca.22407.

If you have any questions, please feel free to contact me on +27 11 717 2204, +27 82-457-0843 or via email at Nicholas.Bacci@wits.ac.za.

If you have any concern over the way the study is being conducted, please contact the Chairperson of this Committee who is Dr Clement Penny, who may be contacted on telephone number 011 717 2301, or by e-mail on Clement.Penny@wits.ac.za. The telephone numbers for the Committee secretariat are 011 717 2700/1234 and the e-mail addresses are Zanele.Ndlovu@wits.ac.za and Rhulani.Mukansi@wits.ac.za

Thank you for reading this Study Information Sheet.

Date: 04 May 2023
