# Supplementary material for: Perceptions of journal editors on the use of eponyms in anatomical publishing: the need for compromise
Source: Anat Sci Int. 2024 Jul 17;99(4):441–53. doi: 10.1007/s12565-024-00789-z (PMC11303421; doi:10.1007/s12565-024-00789-z)
Supplement: Supplementary file 3 — Supplementary file3 (PDF 144 KB) [file 12565_2024_789_MOESM3_ESM.pdf]

Title: Perceptions of journal editors on the use of eponyms in anatomical publishing: the need for compromise

Journal name: Anatomical Sciences International

Authors: Nicholas Bacci*, Erin Hutchinson, Beverley Kramer and Brendon Kurt Billings,

*Corresponding author

Affiliation: School of Anatomical Sciences, Faculty of Health Sciences, University of the Witwatersrand, Johannesburg

Address: School of Anatomical Sciences, University of the Witwatersrand Medical School, Office 2B04, 2nd Floor, 7 York Road, Parktown, Johannesburg, South Africa, 2193

Email: nicholas.bacci@wits.ac.za

Supplementary Information 3

Table A1: Content validity ratios for each questionnaire item and action taken.

Question	CVR	Action
Do you consider eponyms an important aspect of the history of anatomy?	0.667	Retained
Were you aware that since the oldest edition of the Nomina Anatomica (His, 1895), the use of eponymous terms was censured?	0.667	Retained
Are you aware that despite this, new eponymous terms have been coined in anatomy since 1895, and in some medical fields (e.g., Neurology) are still growing?	**0.000**	Amended
In your own opinion, are there valid reasons why eponymous terminology should not be used in anatomy training?	0.667	Amended
n your own opinion, are there valid reasons for the reintroduction of eponymous terminology in anatomical training?	1.000	Amended
Which do you find more useful in peer communication: eponymous terms or Latin-Greek derived anatomical terms?	1.000	Retained
Which do you find more useful in student learning: eponymous terms or Latin-Greek derived anatomical terms?	0.667	Amended
What proportion of eponymous terms acknowledge correctly who contributed to their discovery?	1,000	Amended
Are most eponyms associated with male or female individuals?	**0,333**	Amended
Are you aware of any eponyms related to a person of female gender, at the exclusion of the Hela cells?	**0,000**	Amended
Have you encountered an eponym derived from an individual of Black, Indigenous, or People of Colour in anatomy, at the exclusion of the Hela cells?	**0,333**	Amended
As anatomy and the sciences are becoming more inclusive, do you think that the attribution of new eponyms should be introduced to showcase the emerging diversity across the newly included individuals of diverse backgrounds and genders?	**-0,333**	Amended
If you discovered a novel structure, would you like to see your own name associated with it as a form of acknowledgement of your achievement?	**0,000**	Amended
Do you believe that the removal of eponyms from anatomical terminology would be an effective step towards decolonising the health sciences curriculum?	**0,000**	Amended
Do you believe that the use of the term "Clara cell" is appropriate / should be continued, despite its infamous history?	**0,000**	Amended
Do you believe that the use of the term "Bundle of His" is a0ppropriate / should be continued, despite its infamous association?	**0,000**	Amended
Do you believe mythological eponyms, such as "Achilles tendon", are more acceptable than historical ones?	**-0,667**	Amended
Should eponyms be fully reinstated in official terminology in order to acknowledge the increasing inclusivity and diversity of anatomical and medical fields, particularly in an attempt to address historical injustices?	**0,000**	Amended
Is it ethical to continue using eponymous terms in anatomy?	0,667	Amended

How often do you encounter eponymous terminology in manuscripts received by your editorial office?	1,000	Retained
Do you think the use of eponyms is appropriate in anatomical published work (textbooks, research papers, etc.)?	**0,333**	Amended
Are you aware whether the Terminologia Anatomica 2 (TA2) has restrictions on the use of eponymous terminology?	**0,333**	Amended
In your role as an editor, do you follow the TA2 with regard to the exclusion of eponyms in your journal?	0,667	Amended
Do you think the exclusion of eponyms as prescribed in TA2 is applied by most anatomical journals?	0,000	Amended
Which of the following do you consider valid reasons for the use of eponymous terminology in anatomical publishing?	1,000	Amended
Which of the following do you think are valid reasons for the continued non-use of eponymous terminology in anatomical publishing?	1,000	Amended
Should journals have restrictions on the use of eponyms in their publications?	1,000	Amended
Does the journal of which you are Editor-in-Chief/Senior Editor accept eponym usage in manuscripts?	1,000	Amended
Who, in your opinion, should be responsible for determining whether the recommendations regarding eponyms of the Nomina Anatomica and the subsequent Terminologia Anatomica and Terminologia Anatomica 2 are applied in anatomical publishing?	0,667	Amended
If you believe that eponyms should be restricted in their use, should they be transitioned out of the scientific literature and practice, or be immediately removed from all literature and practical use?	0,667	Amended

Bold values represent questions with low content validity ratio that were amended accordingly.

Table A2: Test-retest results for each questionnaire item.

Question	Test statistic	Df	p-value	Interpretation
Do you consider eponyms an important aspect of the history of anatomy?	McNemar X^2 = N/A	1	N/A	Identical responses between test & retest, perfect match.
Were you aware that since the oldest edition of the Nomina Anatomica (His, 1895), the use of eponymous terms was censured?	McNemar X^2 = 0	1	1	Low statistic and p-value- > 0.05 = no statistical difference between test & retest.
Are you aware that despite this, new eponymous terms have been coined in anatomy since 1895, and in some medical fields (e.g., Neurology) are still growing?	McNemar X^2 = 0.5	1	0.4795	Low statistic and p-value- > 0.05 = no statistical difference between test & retest.
In your own opinion, are there valid reasons why eponymous terminology should not be used in anatomy training?	McNemar X^2 = 0	1	1	Low statistic and p-value- > 0.05 = no statistical difference between test & retest.
n your own opinion, are there valid reasons for the reintroduction of eponymous terminology in anatomical training?	McNemar X^2 = 0.5	1	0.4795	Low statistic and p-value- > 0.05 = no statistical difference between test & retest.
Which do you find more useful in peer communication: eponymous terms or Latin-Greek derived anatomical terms?	McNemar X^2 = N/A	1	N/A	Identical responses between test & retest, perfect match.
Which do you find more useful in student learning: eponymous terms or Latin-Greek derived anatomical terms?	McNemar X^2 = 0	1	1	Low statistic and p-value- > 0.05 = no statistical difference between test & retest.
What proportion of eponymous terms acknowledge correctly who contributed to their discovery?	Mantel-Haenszel X^2 = 0	1	1	H_0 = there is no consistent difference in proportions of answers; p > 0.05 = there is

				no significant difference in answers between test & retest.
Are most eponyms associated with male or female individuals?	N/A			Perfect match, test did not compute.
Are you aware of any eponyms related to a person of female gender, at the exclusion of the Hela cells?	McNemar $X^2 = 0$	1	1	Low statistic and p-value- > 0.05 = no statistical difference between test & retest.
Have you encountered an eponym derived from an individual of Black, Indigenous, or People of Colour in anatomy, at the exclusion of the Hela cells?	N/A			Perfect match, test did not compute.
As anatomy and the sciences are becoming more inclusive, do you think that the attribution of new eponyms should be introduced to showcase the emerging diversity across the newly included individuals of diverse backgrounds and genders?	McNemar $X^2 = 0$	1	1	Low statistic and p-value- > 0.05 = no statistical difference between test & retest.
If you discovered a novel structure, would you like to see your own name associated with it as a form of acknowledgement of your achievement?	McNemar $X^2 = 0.5$	1	0.4795	Low statistic and p-value- > 0.05 = no statistical difference between test & retest.
Do you believe that the removal of eponyms from anatomical terminology would be an effective step towards decolonising the health sciences curriculum?	Mantel-Haenszel $X^2 = 0.0213$	1	0.884	H_0 = there is no consistent difference in proportions of answers; $p > 0.05$ = there is no significant difference in answers between test & retest.
Do you believe that the use of the term "Clara cell" is appropriate / should be continued, despite its infamous history?	McNemar $X^2 = 0$	1	1	Low statistic and p-value- > 0.05 = no statistical difference between test & retest.
Do you believe that the use of the term "Bundle of His" is a0ppropriate / should be continued, despite its infamous association?	McNemar $X^2 = 0.5$	1	0.4795	Low statistic and p-value- > 0.05 = no statistical difference between test & retest.
Do you believe mythological eponyms, such as "Achilles tendon", are more acceptable than historical ones?	McNemar $X^2 = 0$	1	1	Low statistic and p-value- > 0.05 = no statistical difference between test & retest.
Should eponyms be fully reinstated in official terminology in order to acknowledge the increasing inclusivity and diversity of anatomical and medical fields, particularly in an attempt to address historical injustices?	McNemar $X^2 = 0.5$	1	0.4795	Low statistic and p-value- > 0.05 = no statistical difference between test & retest.
Is it ethical to continue using eponymous terms in anatomy?	Mantel-Haenszel $X^2 = 1.0417$	1	0.3074	H_0 = there is no consistent difference in proportions of answers; $p > 0.05$ = there is no significant difference in answers between test & retest.
How often do you encounter eponymous terminology in manuscripts received by your editorial office?	ICC = 0.30 (CI 95% lower bound -1.71; upper bound 0.83)	9	0.30	Poor consistency in responses, vague frequencies can be hard to keep in mind and avoid external recent influences.

Do you think the use of eponyms is appropriate in anatomical published work (textbooks, research papers, etc.)?	McNemar X^2 = 0.5	1	0.4795	Low statistic and p-value- > 0.05 = no statistical difference between test & retest.
Are you aware whether the Terminologia Anatomica 2 (TA2) has restrictions on the use of eponymous terminology?	McNemar X^2 = 0	1	1	Low statistic and p-value- > 0.05 = no statistical difference between test & retest.
In your role as an editor, do you follow the TA2 with regard to the exclusion of eponyms in your journal?	McNemar X^2 = 0	1	1	Low statistic and p-value- > 0.05 = no statistical difference between test & retest.
Do you think the exclusion of eponyms as prescribed in TA2 is applied by most anatomical journals?	N/A			Close to a perfect match, not enough variance in responses for the test to compute.
Which of the following do you consider valid reasons for the use of eponymous terminology in anatomical publishing?	McNemar X^2 = 2.45	1	0.1175	p-value- > 0.05 = no statistical difference between test & retest.
Which of the following do you think are valid reasons for the continued non-use of eponymous terminology in anatomical publishing?	McNemar X^2 = 2.2273	1	0.1356	p-value- > 0.05 = no statistical difference between test & retest.
Should journals have restrictions on the use of eponyms in their publications?	McNemar X^2 = 0	1	1	Low statistic and p-value- > 0.05 = no statistical difference between test & retest.
Does the journal of which you are Editor-in-Chief/Senior Editor accept eponym usage in manuscripts?	McNemar X^2 = 0	1	1	Low statistic and p-value- > 0.05 = no statistical difference between test & retest.
Who, in your opinion, should be responsible for determining whether the recommendations regarding eponyms of the Nomina Anatomica and the subsequent Terminologia Anatomica and Terminologia Anatomica 2 are applied in anatomical publishing?	McNemar X^2 = 0.3	1	0.5839	Low statistic and p-value- > 0.05 = no statistical difference between test & retest.
If you believe that eponyms should be restricted in their use, should they be transitioned out of the scientific literature and practice, or be immediately removed from all literature and practical use?	N/A			Close to a perfect match, not enough variance in responses for the test to compute.

Table A3: Frequency of encountering eponyms in the editorial office

Question	What proportion of eponymous terms acknowledge correctly who contributed to their discovery?	
	N	%
Regularly	1	6.2%
Often	7	43.8%
Sometimes	7	43.8%
Rarely	1	6.2%
Never	0	0.0%
Not sure	0	0.0%
Total	16	100%
